# Editorial: Economic Games, (Dis)honesty and Trust

**DOI:** 10.3389/fpsyg.2022.853653

**Published:** 2022-02-09

**Authors:** Nikolaos Georgantzis, Tarek Jaber-Lopez, Ismael Rodriguez-Lara

**Affiliations:** ^1^Burgundy School of Business, Dijon, France; ^2^Laboratorio de Economia Experimental, Universitat Jaume I Castellon, Castellón de la Plana, Spain; ^3^EconomiX, University Paris Nanterre, UPL, Paris, France; ^4^Teoría e Historia Económica, Campus de la Cartuja, University of Granada, Granada, Spain; ^5^Teoría e Historia Económica, Campus El Ejido, University of Malaga, Malaga, Spain

**Keywords:** trust, honesty and dishonesty, prosocial behavior, anti-social behavior, trustworthiness

Trust is a central source of well-being in a society. When individuals feel that they can trust others, cooperative interactions become more likely, making a group of individuals able to enjoy better outcomes than the sum of individual stand-alone efforts would achieve. Opportunistic and dishonest behavior hinders trust by generating negative feedback to trusting behavior. In this Research Topic we collect cutting edge research on pro-social behavior, trust, and (dis)honesty. Below, we offer a brief discussion of the article included, under two general headings: (i) trust and trustworthiness and (ii) dishonesty and opportunistic behavior.

## Trust and Trustworthiness

Does the emergence of a crisis mitigate or substitute people's concerns regarding social issues? Blanco et al. suggest that donations aimed at addressing other social concerns are partially substituted by donations to COVID-19 funds. Yet, this substitution does not fully replace all other social concerns. Trusting the charitable organization is the most important factor to explain donations to a charity. These findings imply that the COVID-19 pandemic may substitute other social concerns, highlighting the importance of trust toward charitable institutions.

Which other societal factors foster trust in a society? Three contributions address the role of societal factors like culture, inequality, and social class in the emergence of trust. Rodrigo-González et al., find that inequality is an important explanatory factor of trust. In a trust game, trustors send more to those who have a higher endowment, probably under the belief that better performing people are more trustworthy. Trustees reciprocate more toward trustors who are richer when their money is determined by their effort. There is also evidence that trustees reciprocate more when they observe the history of decisions, and particularly trustor accumulated profits from past actions. Zylbersztejn et al. employ the hidden action game in Charness and Dufwenberg (2006) in two different locations, France and Japan. In both settings, observers are asked to predict the behavior of trustees in the hidden action game, after watching a mugshot picture or a muted video of the trustees, making a non-strategic statement independent of the hidden action game, or a loaded video in which the trustee made a strategic pre-play statement in front of the trustors. Their results suggest that observers account for morphological traits of the trustees and this bias persists across cultures. They also show that cultural distance is not *per se* helpful or detrimental for predicting trustworthiness. Rather, it affects ways in which people exploit observable information in social interactions. Finally, Qiang et al. find that social class may affect trust, but they also show that a social class-specific perception of control may be a mediating psychological mechanism in the association between social class and trust beliefs. Specifically, members of the upper social class are inclined to perceive high control over their outcomes, and they have a strong trust in daily life, while members of the lower social class are more likely to feel a low sense of control, and in turn, low social trust. Focusing on another individual driver of cooperative behavior and trust, in a lab-in-the-field experiment with prison inmates, Balafoutas et al. investigate whether there is a connection between psychopathy and pro-sociality. They find that psychopathy correlates with anti-social behavior in its various forms, like weaker reciprocity to trust (trustworthiness), lower cooperation, lower benevolence, and more bribing.

## Dishonesty and Opportunistic Behavior

In order to improve our understanding of the determinants of cheating behavior and expectations about it, the following contributions address the role of the emotional state, gender and the environment in the emergence of dishonest behavior.

Medai and Noussair induce emotional states to participants by asking them to watch a video prior to rolling a die. The authors consider two different treatments, depending on whether or not the video induces a positive emotional state (Happiness) or does not have any effect on emotional state (Neutral). The main result of their paper is that the level of dishonesty (opportunistic misreporting of the die rolling task) is lower in the Happiness treatment, compared with the Neutral treatment. They further argue that there are no differences in lying behavior when looking at the behavior of men and women. A further examination of gender differences in lying behavior is pursued by Muñoz García et al.. In their article, they employ a modified die-under-the-cup task, in which the experimenter can observe the real distribution of the rolls. They find gender differences in cheating behavior in that women are satisfied with lower earnings than men. The frequency of radically dishonest subjects (those who did not even roll the die) is larger among men, while the proportion of “lucky honest” (rolling, but misreporting) is larger among women. Gender differences are also reported by Monzani et al., who study the drivers of anti-social behavior among entrepreneurs. Their results revealed that displaying authentic leadership reduced the likelihood of entrepreneurs (vs. managers) and men (vs. women) of engaging in antisocial behaviors such as lying to harm one's competition or seeking an unfair advantage by cheating.

Pascual-Ezama et al. find that different types of cheaters exhibit different abilities to detect unethical behavior. In their online experiment, participants are shown videos from Golder Balls, one of the most popular TV shows in the UK and they are asked to predict whether or not contestants will be dishonest. Their participants do not beat randomness in detecting dishonest behavior, but some types of cheaters are better at detecting honesty than others. The authors also highlight the importance of (non-)verbal cues and information to detect unethical behavior, and provide evidence of a “preconceived honesty bias” (i.e., people tend to think that honesty prevails). Chapkovski et al. in a sequential version of the die-rolling task, find that the likelihood to cheat increases in a “collaborative” setting, in comparison with an individual one. As the game is repeated across 45 rounds, participants become more dishonest over time in the collaborative treatment, whereas there is no such trend in the individual condition.

In a tax-evasion experiment, Du et al. randomly assigned a gross income to be declared to a central tax authority. One of the subjects in the group is randomly selected in each round to be audited. If the subject has misreported his/her income, then she will need to pay a fine. A whistleblowing mechanism is shown to be effective in both curbing tax evasion and improving the precision of tax auditing. In addition, the authors find no evidence of spillover effects of whistleblowing on ingroup cooperation in the subsequent generalized gift exchange game.

Finally, in a theoretical contribution, Spiegelman addresses academic dishonesty in the presence of open data practices. A signaling model is presented to show that both high- and low-quality results may be published in both open and closed data regimes, but open data is favored by high-quality results. A measure of “science welfare” is proposed, to show that open data will always improve the aggregate state of knowledge.

## Author Contributions

All authors listed have made a substantial, direct, and intellectual contribution to the work and approved it for publication.

## Conflict of Interest

The authors declare that the research was conducted in the absence of any commercial or financial relationships that could be construed as a potential conflict of interest.

## Publisher's Note

All claims expressed in this article are solely those of the authors and do not necessarily represent those of their affiliated organizations, or those of the publisher, the editors and the reviewers. Any product that may be evaluated in this article, or claim that may be made by its manufacturer, is not guaranteed or endorsed by the publisher.
